# Is reliance on an inaccurate genome sequence sabotaging your experiments?

**DOI:** 10.1371/journal.ppat.1007901

**Published:** 2019-09-12

**Authors:** Rodrigo P. Baptista, Jessica C. Kissinger

**Affiliations:** 1 Center for Tropical and Emerging Global Diseases, University of Georgia, Athens, Georgia, United States of America; 2 Institute of Bioinformatics, University of Georgia, Athens, Georgia, United States of America; 3 Department of Genetics, University of Georgia, Athens, Georgia, United States of America; McGill University, CANADA

## Abstract

Advances in genomics have made whole genome studies increasingly feasible across the life sciences. However, new technologies and algorithmic advances do not guarantee flawless genomic sequences or annotation. Bias, errors, and artifacts can enter at any stage of the process from library preparation to annotation. When planning an experiment that utilizes a genome sequence as the basis for the design, there are a few basic checks that, if performed, may better inform the experimental design and ideally help avoid a failed experiment or inconclusive result.

## All genome sequences have “issues”

There are many factors that can affect the ultimate genome sequence and annotation that are produced, and both should be considered “works in progress.” An awareness of these factors can inform experimental decisions that may depend upon the accuracy of a particular genome sequence, region, gene, or genes. This Pearl focuses on eukaryotic sequences, as they are still subject to greater challenges, but many factors are universal. The target audience for this Pearl is the wet-bench researcher who can use web-based genome resources, commercial genomics software, or has access to a member of the community with a small amount of genomics or bioinformatics experience. No coding is required to address the questions and solutions that are proposed.

### What is the origin of the sample used to generate the genome sequence?

The origin matters. Did the sample originate from a clone, a mixed population (common with microbes), or possibly a hybrid? Differences between individuals can be single nucleotide polymorphisms (SNPs), but often they involve insertions or deletions (indels) of various sizes, copy number variations (CNV), and even small rearrangements. Hybrids can have dramatic differences between orthologous chromosomes [1]. Genome sequences derived from a heterogenous population, especially when CNVs exist, complicate genome assembly, and often the sequence produced is a composite of the major alleles present in the sequenced sample. Genome sequences derived from clonal laboratory strains are often easier to assemble, but they may not be truly representative of circulating wildtype strains because they are adapted to culture and, if propagated for a long time, may have lost genes or accumulated mutations [2].

Knowing the ploidy of your organism, especially if it is subject to aneuploidy, and the ploidy of the source material (e.g., haploid gametes versus diploid tissue) that was sequenced can help inform many situations, such as variation at a site being homozygous or heterozygous. Knowledge of the ploidy of the sequenced material helps with estimates of CNV. It is always advisable to experimentally confirm regions of a genome sequence in your organism/stock/strain if your experiment will depend upon the veracity of that sequence.

### Does the genome have troublesome characteristics?

**Fig 1:**
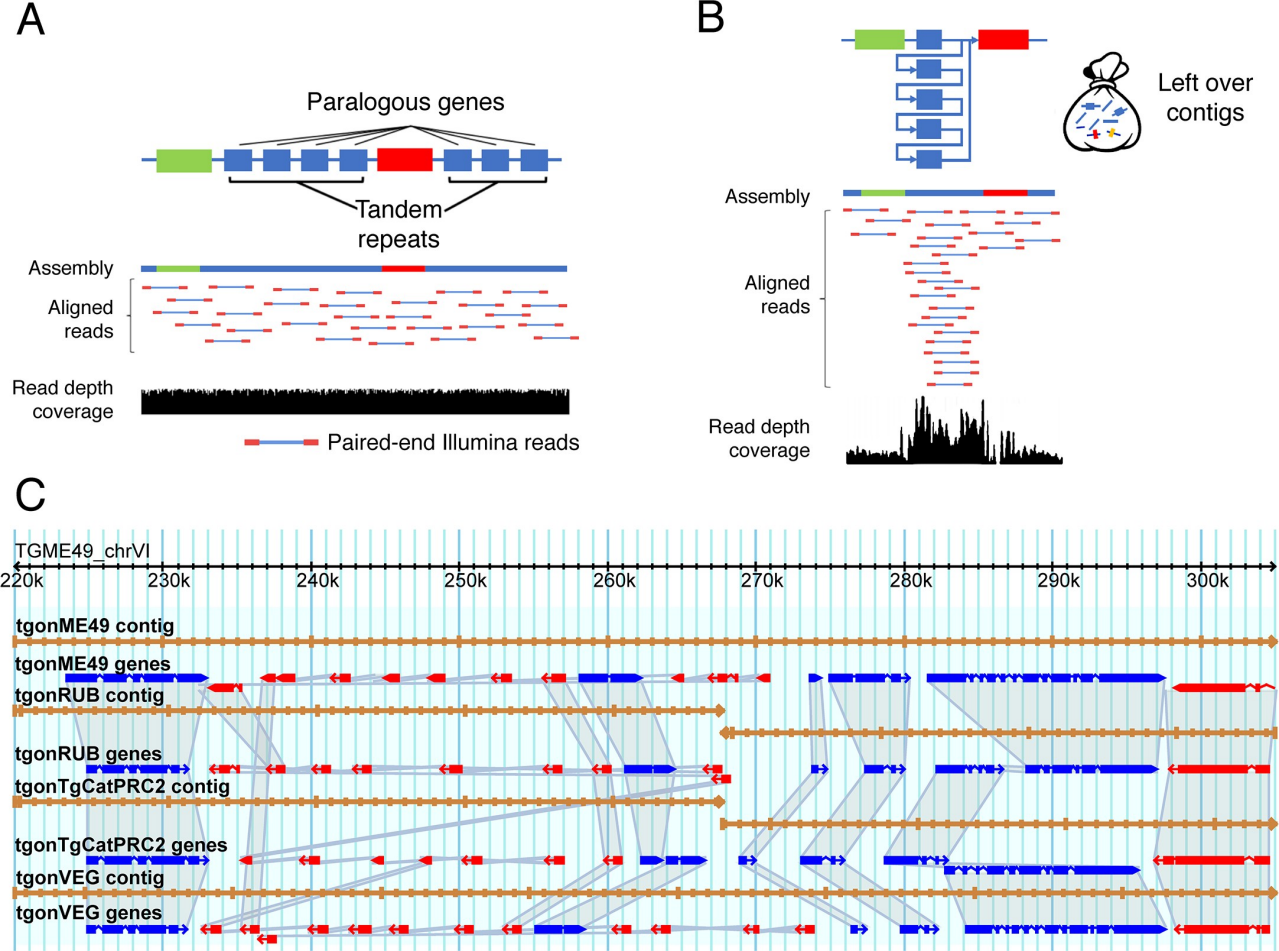
Common genome assembly problems. (A) Expected genome organization with roughly equal distribution of aligned reads across the genome sequence. (B) Illustration of a collapsed repeat region and detection via an accumulation of mapped reads resulting in a peak region in the depth coverage plot. (C) An 85-kb region shown for four strains of *Toxoplasma gondii* chr VI. Contiguous reads are shown as yellow and green horizontal lines. Annotated genes are shown in blue (forward strand) and red (reverse strand). Grey shading indicates orthology. The region defined by the orange window near 270-kb mark (top ruler) highlights the gap in contigs for two strains likely caused by the repetitive surface antigen genes located in the 238–275-kb region.

Some genome sequences are physically difficult to sequence because of extreme nucleotide bias. The *Plasmodium falciparum* genome sequence was so AT-rich that specialized sequencing chemistry was developed [3]. Long homopolymeric runs of any base are particularly troublesome for some sequencing technologies [4] and may lead to an incorrect number of nucleotides, resulting in frame-shifts if the sequence is coding. If a putative frameshift interrupts your gene of interest, confirm its presence in your stocks with PCR and Sanger sequencing, ideally, or view the assembly (see Fig 1) before accepting it. If the genome sequence contains numerous repetitive sequences, retrotransposons or mobile elements, or large, highly similar gene families, the genome assembly will be affected (Fig 1), especially if only short-read sequences were used.

Repetitive sequences are a huge challenge for most assembly algorithms. It is well established that multiple variant copies of genes can increase novelty and help a pathogen to survive in the face of immune system pressures [5] and are often related to pathogenesis and virulence [6–8]. Long-read and single-molecule technologies like PacBio and Nanopore [9] can provide verification of tandem copies and in some cases, the number of gene copies. Low-coverage, less accurate, long-molecule reads can be used as a framework upon which shorter-read sequences can be mapped, or long reads, when sufficiently deep, can be used for the complete assembly and provide self-error correction [10–12].

There is an easy way to assess the quality of your organism’s genome assembly. Map the reads from the sequencing project back to the assembled genome sequence and have a look (Fig 1A) (see the following sections for pointers on how to do this: “How as the genome assembled?”, “How good is the assembly?”, “Was the assembly corrected?”, and “Common challenges and strategies to help”). This quick screen checks for “pile-ups,” the tell-tale indicator for the presence of collapsed repetitive sequence regions in the assessed genome sequence (Fig 1B). Alternatively, a genomic Southern blot utilizing a restriction enzyme that cuts once within your sequence of interest will also reveal additional copies if present. The reference genome assembly for the apicomplexan parasite *Toxoplasma gondii* ME49 contains several collapsed regions that vary by strain (Fig 1C) [8]. Despite the high quality of this genome sequence and its correspondence to genetic maps, issues related to the number of chromosomes still exist [13, 14].

### How were the libraries prepared?

Before long-read technologies existed, large distances were covered by biological libraries of different insert sizes in plasmids, bacterial artificial chromosomes (BACs), cosmids, and fosmids. Generation of single reads from each end of a known length (e.g., 10 kb) library insert sequence would suggest that the reads should end up in the assembled genome facing each other and about 10 kb apart. If they are not, it is suggestive of an assembly error. Genome sequences that relied on cloning and biological replication have additional issues that need to be considered. Some sequences simply cannot be cloned; they are toxic to the organism used for cloning and replication and thus, will be missing in the genome sequence produced. Unclonable sequences often contain a few select genes and heterochromatin. The inverse is also true; a DNA sequence from the cloning vector or organism used to construct the library can end up in the assembled target genome sequence. Next-generation sequencing (NGS) approaches don’t suffer from this particular problem as sequences are not propagated in live organisms, but the trade-off is a loss in read length size, down to 50–150 bp down from the approximately 1 kb produced by Sanger chemistry. Some of the newest generation technologies (see the following section, “What sequencing platform was used?”) do not have this issue.

High-throughput NGS library preparation plays a critical role with respect to the quality of the genome sequence produced. Many protocols contain amplification steps, which can introduce bias. For example, single cells can be used for genome sequencing but via the application of whole genome amplification (WGA). The approach is powerful when material is limited, but the amplification process is biased, and several different WGA reactions (on different cells or populations of like cells) are necessary to fully identify and remove the amplification bias [15, 16]. It should be noted that bias is rarely removed from the reads submitted to archives, so it is imperative to know if WGA was utilized.

### What sequencing platform was used?

Different sequencing platforms have different strengths and weaknesses [9], and they continue to evolve rapidly and often complement each other if several different approaches are applied. Genome sequences assembled with Sanger chemistry will have good quality sequence, but the assembled genome sequence will be affected by the library issues mentioned previously. Genome sequences generated with legacy systems, e.g., 454 and Ion Torrent, will have homopolymer miscount issues. Newer genome sequences will consist of highly accurate Illumina short-read technology, but the assembled sequence, especially if repeats are present, will be incomplete and contain gaps and mis-assemblies unless a hybrid assembly using long-read technologies like PacBio or Oxford Nanopore are utilized.

### How was the genome assembled?

Sequence assemblies are of two types: de novo, assembled from scratch, and reference-based. The latter is normally used when an established organismal reference genome already exists and the experimental goal is to determine variation with respect to it. It is not a good approach to detect rearrangements or syntenic breaks, but it is ideal to detect SNPs, some indels, and CNV. Reference-based approaches will not reveal genome features not present in the reference, a significant drawback. Due to the large volume of population studies focused on SNPs, most genome sequence data, sadly, remain as unassembled files of reads.

De novo assemblies are the only option for an organism’s first genome sequence, and when possible, they should be performed as a matter of practice to permit discovery of new features. In the case of eukaryotic genome sequences, especially when the karyotype is unknown and physical maps do not exist, reads can only be partially assembled into contiguous reads, “contigs,” or scaffolds of contigs, containing gaps. Contigs often contain sequences that are fairly unique because repetitive sequences are often “masked” in a de novo assembly because of the issues they cause. As a result, contigs often end at, or are separated by, missing repetitive regions that were not utilized (e.g., masked) or could not be resolved during the assembly. Variation found at the ends of contigs should be treated with caution.

Gaps between contigs that have been ordered and oriented into scaffolds are often indicated by exactly 100 “N’s” to indicate a gap of unknown size. In some cases, scaffolds representative of whole chromosomes are assembled, but these, too, often contain numerous gaps or ambiguous bases (Table 1). Some assemblers also create a scaffold that links together all “leftover” contigs. Beware of this scaffold, often named “scaffold 0,” as the order and orientation of these contigs bears no resemblance to their biological location; it is simply a convenient mechanism to make sure all contigs are available to those using or searching the genome sequence.

**Table 1:** Genome sequence status of common eukaryotic hosts and pathogens.

Species	K^a^	Genome Assembly Status				Initial Release	Most Recent	
		Scaffolds	Gaps	N’s^b^	Genome Size (MB)		Assembly	Annotation
**Fungi & Oomycetes**								
*Aspergillus fumigatus* Af293	8	8	11	575,000	28.75	2005	2005	2019
*Candida albicans* SC5314	8	8	80	6,259	14.28	2004	2016	2018
*Candida auris* 6684	7	99	660	165,810	12.49	2015	2015	2017
*Coccidioides immitis* RS	4	7	4	400	29.01	2004	2015	2015
*Cryptococcus gattii* WM276	14	14	8	13,078	18.37	2011	2011	2011
*Fusarium graminearum*	4	31	414	234,405	36.45	2003	2017	2017
*Fusarium oxysporum* 4287	15	114	1,277	1,450,151	61.38	2007	2015	2015
*Histoplasma capsulatum* NAm1	-	280	2,592	2,381,013	33.03	2005	2014	2014
*Magnaporthe oryzae*	7	53	163	29,800	40.97	2003	2016	2016
*Paracoccidioides brasiliensis* Pb18	5	57	499	483,815	29.95	2008	2008	2014
*Phytophthora infestans*	-	4,921	13,367	38,410,029	228.54	2006	2014	2014
*Phytophthora ramorum*	-	295	1	214	60.25	2006	2018	2018
*Pneumocystis jirovecii* RU7	-	70	0	0	8.39	2015	2015	2015
*Saccharomyces cerevisiae* S288C	16	17	0	0	12.16	1999	2014	2018
**Plant hosts**								
*Arabidopsis thaliana*	5	7	95	185,644	119.66	2001	2018	2019
*Oryza sativa*	12	58	256	117,485	374.42	2002	2015	2018
*Zea mays* B73	10	598	2,522	30,732,878	2,135.08	2010	2017	2017
*Triticum aestivum*	7	22	692,976	275,682,619	14,547.26	2017	2018	2018
**Vertebrate and other hosts**								
*Homo sapiens*	23	473	875	151,122,679	3,099.73	2002	2015	2019
*Mus musculus*	21	162	634	78,088,216	2,730.85	2004	2017	2017
*Gallus gallus*	35	525	946	9,784,460	1,065.36	2004	2018	2018
*Anopheles gambiae*	3	8,145	8,735	12,572,948	265.02	2002	2014	2018
*Ixodes scapularis*	15	369,492	201,145	376,910,010	1765.38	2008	2012	2017
**Protists**								
*Acanthamoeba castellanii* Neff	-	384	2,808	2,576,247	42.01	2013	2013	2014
*Babesia bovis* T2Bo	4	13	0	0	8.17	2007	2007	2007
*Cryptosporidium hominis* TU502	8	358	7	119	8.91	2004	2004	2013
*Cryptosporidium parvum* IOWA II	8	8	10	14,600	9.10	2004	2007	2018
*Cyclospora cayetanensis* CHN_HEN01	-	2,297	1,276	71,547	44.03	2016	2016	2016
*Eimeria tenella* Houghton	14	4,665	8,063	686,045	51.89	2013	2013	2015
*Entamoeba histolytica* HM-1:IMSS	-	1,529	643	64,300	20.83	2005	2005	2014
*Giardia lamblia* isolate WB	-	92	214	21,400	11.21	2007	2007	2014
*Leishmania major* Friedlin	36	36	0	7	32.85	2005	2005	2019
*Plasmodium berghei* ANKA	14	100	123	126,523	18.56	2014	2014	2019
*Plasmodium falciparum* 3D7	14	15	0	0	23.32	1998	2016	2019
*Plasmodium vivax* P01	14	242	340	137,629	29.04	2013	2018	2018
*Sarcocystis neurona* SN3	-	871	2,320	1,981,126	124.40	2014	2014	2015
*Theileria annulata* Ankara	4	8	2	200	8.35	2005	2005	2015
*Toxoplasma gondii* ME49	14	2,277	244	203,077	65.66	2008	2013	2015
*Trichomonas vaginalis* G3	-	64,769	8,181	818,393	176.42	2005	2005	2014
*Trypanosoma brucei brucei* TREU927	11	12	39	3,590	26.07	2005	2005	2019
*Trypanosoma cruzi* CL Brener	41	29,495	3,251	325,100	89.93	2005	2005	2019

Data obtained from NCBI Genbank and EuPathDB.org, Release 41, Dec 2018.

^a^ Karyotype

^b^ Ambiguous bases

“-”Unknown

Know your analysis goal before choosing an assembly strategy. Many times, an assembly is not even necessary, and this will save time and money. If a reference genome sequence is already available, you can use unassembled reads to detect sequence variants and CNVs much faster without assembly.

### How good is the assembly?

There are ways to define the quality of the assembly, usually by calculating its contiguity. The statistics used are N50 and L50. If the scaffolds and contigs are ordered from largest to smallest, the N50 is the sequence length of the shortest contig in the list at the point where 50% of the total genome length is present above it. The larger the number, the more contiguous the assembly. L50 is smallest number of contigs whose length sum makes up half of genome size. Thus, a smaller number is better. Gaps are another useful metric. A fully assembled genome sequence will have no gaps and a number of scaffolds that is equivalent to the number of chromosomes and ideally contain telomeres.

Each type of sequence assembly comes with a set of inherent issues, and most genome sequence projects produce an assortment of leftover reads and contigs that do not assemble. In some cases, these reads can be identified as contamination, an unexpected symbiont, or organellar genome sequence. In other cases, the leftover bits are a tell-tale sign of particular types of assembly errors or unexpected genome sequence variation, e.g., CNV (Fig 1) or high levels of heterozygosity between alleles (especially if a population was sequenced, rather than an individual). New ploidy-aware assembly programs are emerging, and they will assist greatly with several of the issues presented here. For this reason, it is important to know when and how a genome sequence was assembled (Table 1). Remember, a genome sequence can always be reassembled from the archived reads as new algorithms and new, or longer, sequences become available. Many communities are actively reassembling important reference sequences.

Leftover individual sequence reads are rarely deposited as part of the genome record, but unscaffolded contigs >2 kb are usually deposited as contigs in addition to the scaffolds, or as scaffold 0, and will be searchable via BLAST. All sequence reads from a genome project can be obtained from read archives like the short-read archive (SRA) [17]. If there is a need to validate a particular SNP or indel, this can be done experimentally with PCR and Sanger sequencing or by viewing the reads mapped in the assembly [18–20], including the number of reads mapped to each strand of the genome sequence, which can provide support, or not, for a particular variant. Multiple reads mapping to both strands of the genome sequence should be present if the variant is true.

### Was the genome sequence “corrected,” and if so, how?

Error-prone long-sequence reads can be corrected prior to assembly using proovread [21]. Correction prior to assembly can facilitate assembly when the error rate is high, e.g., in low-coverage PacBio reads. Assembled genome sequences can also be “polished.” Polishing involves base call correction, and ICORN2 [22] is a popular tool. Polishing is performed using highly accurate Illumina reads mapped back against the final genome assembly. Read correction and polishing are useful and recommended steps, but they are highly dependent on the performance of the aligner, and the end user must be aware that the corrected and polished sequences will represent the most abundant alleles present in the reads. In other words, isoforms and rare variants of repetitive sequences will be “corrected,” i.e., overwritten, in the final assembly by more abundant sequence variants.

### How was the assembled sequence annotated?

Annotations can be *ab initio* (determined algorithmically based on statistical properties), evidence based, or, most often, a combination of the two approaches. Older annotations, or annotations of evolutionarily distant, hard to obtain or grow organisms, tend to be *ab initio* as there was, or is, often a lack of expressed sequence tag (EST), RNA-Seq, proteomic, or synteny information available as evidence. Currently, many gene prediction tools are widely used by the community [23], and some are combined in Maker2 [24]. *Ab initio* prediction tools, when combined with external data such as orthology with a closely related species, RNA-Seq, and Mass Spec peptides, leads to an increase in accuracy of predicted gene features [24, 25]. As additional RNA expression data became available for *Cryptosporidium parvum*, it was shown that the number of introns was significantly underestimated [26]. The addition of the newly discovered introns altered the predicted protein sequences and the structure of numerous genes.

Gene predictions are genome-assembly dependent, which means if a region is missing, it cannot be annotated. Likewise, if the region is poorly assembled or missing in a reference genome sequence used for orthology, it may end up missing in the genome sequence that is being annotated. A good example is *Cryptosporidium*. The genome sequence for *C*. *parvum* was released in 2004, with a state-of-the-art assembly and annotation for the time [27]. This genome sequence was used as the reference sequence for several additional *Cryptosporidium* strains and species [28, 29]. This practice can be dangerous, as one of the genome features that facilitates speciation is genome rearrangement, which affects chromosome pairing during reproduction. As there are no genetic systems for many pathogens that can be used to generate a physical map, reference mapping is useful, but it is easy to forget the origins of genome sequence assemblies and annotation created or propagated in this way, so care must be exercised when using reference-mapped genome assemblies as the basis for experiments.

## Common challenges and strategies to help

A few relatively quick checks of the state of your organism’s genome sequence can save time, expense, and frustration with experiments that “should work” but don’t.

### The gene is annotated as single copy, is it?

Additional copies of genes can thwart experiments designed to target, clone, delete, or modify a particular gene. The annotation may indicate a single-copy gene, but depending on the technology used to generate your genome sequence, nearly identical copies of genes can become assembled as one gene (short-read only assemblies are most prone to this issue), and slightly divergent gene family members, especially if they are in tandem repeats, often don’t assemble and can be found in the leftover reads or small unassembled contigs (Fig 1). Quick checks for read depth around the gene of interest (as described previously) can confirm if this is a potential issue.

### The annotation doesn’t describe your gene. Is it really missing from the genome?

It is easy to be misled on the basis of existing annotation that a gene is missing. Genes can be lost, and they do decay or evolve beyond recognition, but they may also be missing because of a sequence assembly gap. Missing genes, especially if they are part of a gene family, can often be located in the unassembled reads or contigs (Fig 1B). Note that unassembled contigs are often not annotated, so they should be searched using BLASTX (protein against translated nucleotides). The best practice for determining gene loss is to look at a synteny map of the genomic contigs and see if the region of the genome that is expected to contain the gene of interest (based on its location in a close species) is present, conserved, and not rearranged (Fig 1C). Alternatively, the region may be missing from the genome assembly, i.e., a gap relative to the comparator sequence. Misassemblies and gaps can provide the illusion of missing genes, when in reality, they are missing from the assembly, have evolved into pseudogenes, or, in some cases, have been replaced by a horizontal gene transfer located elsewhere in the genome.

Genome sequence gaps have many downstream consequences. The number of genes may be reduced relative to the actual number, and ironically, the number of genes can also be inflated because a portion of the same gene can be found on each side of the gap, resulting in two partial predictions. Small assembly gaps often lead to frameshifts in coding sequences, which, in turn, lead to an artificial increase in the number of pseudogenes, when, in reality, the culprit is an assembly gap. Gaps can also indicate the location of a missing tandem array of genes or repeat sequences that could not be properly assembled (Fig 1C).

### Can I trust the annotation?

Some organismal genome sequences are continuously curated by the community or experts and have a good, recent genome annotation (Table 1). However, annotators cannot annotate what does not exist (e.g., gaps). Eukaryotic genome sequences, especially from animal, vector, or plant hosts, are complex, and even with continuous curation, there is much more to be fixed and discovered as new sequence technology, assembly algorithms, and experimental evidence appear. For example, untranslated regions and noncoding RNAs aren’t routinely annotated. All genome sequences and their annotation are “works in progress” and are static representatives of one point in time for a continuously evolving molecule within a genetically diverse population.

### Does the annotation affect pathway analyses?

Yes. Studies aimed at drug target discovery often look for a gene that appears to be essential to a pathway. Once discovered, the gene is knocked out, and to everyone’s dismay, it was not essential, and the organism survives in the presence of drug. There are many reasons this may have happened, which range from the ability of the drug to reach the target to the possibility that the assessment of essentiality is flawed. Errors in the annotation or the assembly can also lead to this result. For example, the gene may not be single copy, or the knockout construct behaved oddly and targeted a related or additional gene copy of the target, producing unusual or hard to interpret results. Alternatively, the large proportion of genes of unknown function (as high as 40% in some organisms) encode functions that allow the organism to circumvent the knockout. Much work is still needed on this important class of genes.

## Conclusions

### Take a little time to confirm the target(s) of your experiments

Many of the pitfalls described here can be easily avoided with a few simple experiments and knowledge of your genome sequence (Table 1). It is always advisable to PCR and sequence a target gene prior to use in order to validate gene presence, sequence, variation or homopolymer tract lengths, and frameshifts. While it is very difficult to prove a gene does not exist, the checks described previously can help to rule out the possibility it is missing because of gaps in the genome sequence. A simple BLASTX search of the entire genome sequence with a related protein sequence of interest can identify sequences or partial sequences that can encode related proteins even if they were not annotated or reside on the unassembled contigs.

It is also advisable to check for additional copies of the gene if their presence will matter in the context of your experiment. A personal computer can easily map reads to a reference genome to look for pile-ups indicative of copy number issues (Fig 1B) that may affect your gene of interest. Desktop applications like Geneious [30], among others, have the appropriate tools to map reads. It is also important to check your community databases, for example, EuPathDB.org [31], GeneDB.org [32], Gramene [33], or VectorBase.org [34], among others, that work directly with the community, have trained curators, and provide the most up-to-date sequence annotation. Finally, contact the members of the community who generated or are actively working on the genome sequence. They can be a tremendous resource for insight into any issues you may be experiencing and will welcome input on problematic areas you have experimentally resolved.

### There is hope

Newer sequencing platforms that generate longer reads, such as PacBio and Nanopore, are helping to resolve many tricky but important tandem gene problems and are closing gaps in genome sequences where they have been applied. Some genome sequences will require additional approaches beyond long reads, such as Hi-C (chromatin conformation capture) [35], Chicago library methodologies [36], or optical mapping [37]. Truly difficult genome sequences can be hexaploid (like wheat), have enormous numbers of scaffolds (like *Ixodes scapularis*, which has >350,000), be littered with highly similar repeat elements (like *T*. *vaginalis*), or suffer from extreme heterogeneity and length differences between sister chromosomes (as in the hybrid *T*. *cruzi*). Some genome sequences have already been “fixed” with these new technologies, but there is still significant work required to make them as good as they can be. New assemblies and annotations are always needed. It is frustrating when all the naming and numbering changes, but these changes result from progress that will facilitate and inform the basis of much-needed further experimentation.
